# A Case of Ruptured Left Interstitial Ectopic Pregnancy

**DOI:** 10.7759/cureus.45711

**Published:** 2023-09-21

**Authors:** Snehal Deshmukh, Sonali Shelke, Deepti Shrivastava

**Affiliations:** 1 Obstetrics and Gynaecology, Datta Meghe Medical College, Datta Meghe Institute of Medical Sciences (DU) Wardha, Nagpur, IND; 2 Obstetrics and Gynaecology, Jawaharlal Nehru Medical College, Datta Meghe Institute of Medical Sciences (DU) Wardha, Nagpur, IND

**Keywords:** laparotomy, cornual resection, cornual, ectopic pregnancy, interstial

## Abstract

Although rare, interstitial ectopic pregnancy poses a challenge in diagnosis and management. The pregnancy is implanted in the interstitial part of the fallopian tube, i.e., the proximal intramural portion. When ruptured, it results in a catastrophic event; the rupture can involve the uterine wall, as in this case, which puts the prognosis of future pregnancies at risk. Here, a case of a 35-year-old primigravida who conceived after ovulation induction is reported. Her diagnosis of ectopic pregnancy was missed; it was misdiagnosed as incomplete abortion followed by dilatation and evacuation. Post-evacuation severe pain and hemodynamic instability, and subsequent ultrasonography (USG) lead to the diagnosis of left interstitial ectopic pregnancy. Emergency laparotomy, left salpingo-oophorectomy, and cornual resection with repair were done.

## Introduction

An interstitial pregnancy (IP) is defined as "when the blastocyst implants within the myometrium in the most proximal section of the fallopian tube (called the interstitial portion)." Interstitial ectopic pregnancy is a rare ectopic pregnancy, constituting 2%-6% of all ectopic pregnancies. It has a high mortality. Some literature considers cornual and interstitial as similar entities, but interstitial refers to blastocysts implanted inside the proximal intramural region of the tube, whereas cornual implantation characterizes those in the upper and lateral uterine cavity. The management of such pregnancies depends on the time of presentation to the hospital, time of diagnosis, size of pregnancy, location of pregnancy, obstetrics history, and any predisposing factors [1,2]. Rare chances are there of misdiagnosing such pregnancy or failure to diagnose at all. This case report is a rare presentation of misdiagnosing interstitial ectopic pregnancy as incomplete abortion, but getting timely treatment after the correct diagnosis can definitely save the patient's life. A high degree of suspicion will always help in landing at the correct diagnosis.

## Case presentation

A 35-year-old female patient was referred to our hospital from an outside rural hospital. The patient had taken ovulation induction drugs as she had infertility of three years. After missing her periods for about two months, she got a home urine pregnancy test that showed a positive result. The patient did not confirm her pregnancy by visiting any doctor or getting an ultrasonography (USG). After two weeks, she had pain in the abdomen with vaginal bleeding. She consulted a doctor, and ultrasonography was done, which suggested incomplete abortion. The patient underwent dilatation and evacuation. After 24 hours of dilation and evacuation, the patient complained of severe pain in the abdomen and was referred to our hospital.

**Figure 1:**
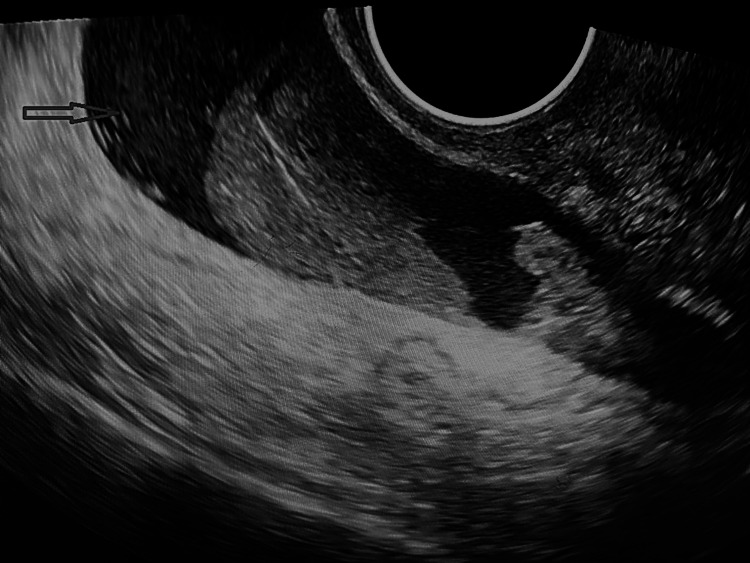
Gross hemoperitoneum evident on ultrasonography (arrow)

**Figure 2:**
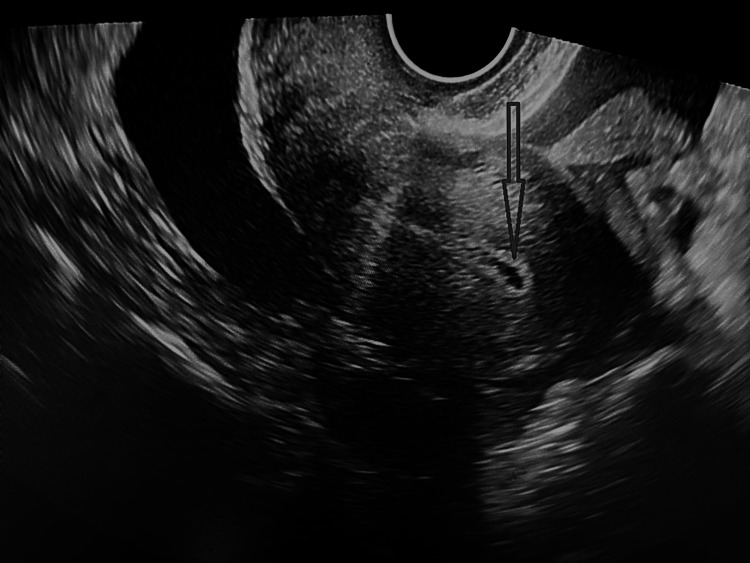
No intrauterine gestational sac and presence of gross hemoperitoneum (arrow)

At the time of presentation, the patient was pale. She was in agony due to pain. On examination, she had tachycardia of around 120 beats per minute, and her blood pressure was 90/60 mmHg. On abdominal examination, she had tenderness and guarding. The patient had fornicial tenderness even on per vaginal examination. On emergency ultrasonography, gross hemoperitoneum was present (Figure 1), with an empty uterus (Figure 2). The right ovary and fallopian tube were normal. The left ovary was normally visualized. There was no typical gestational sac (G sac) seen in the fallopian tube or anywhere. In view of hemoperitoneum and preceding history, the decision for laparoscopy was taken, but due to surgical inexpertise, it was converted to laparotomy.

**Figure 3:**
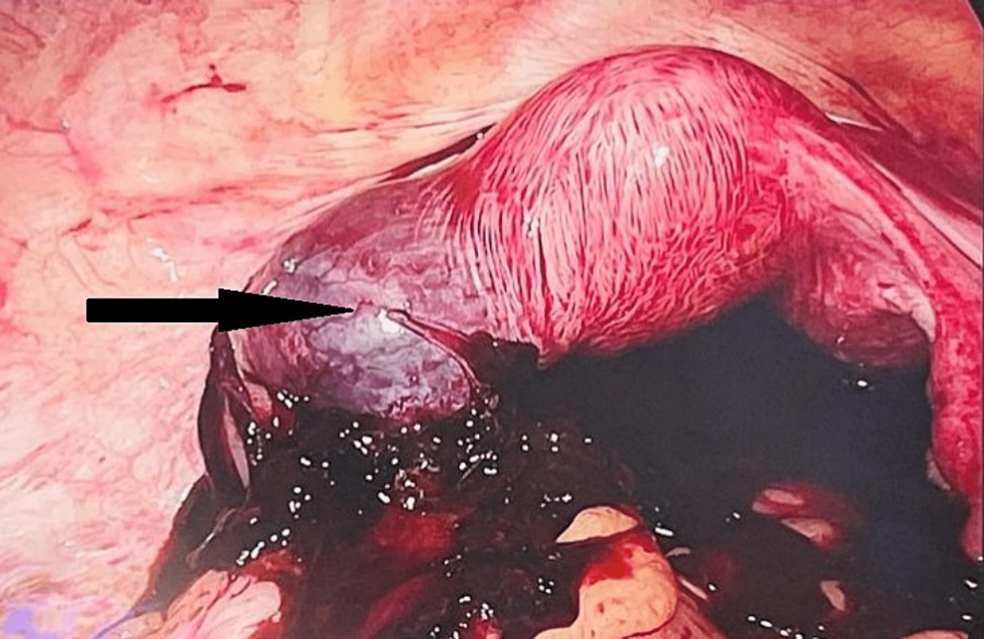
Ruptured left interstitial (cornu) ectopic pregnancy (arrow)

Intraoperatively, a hemoperitoneum of 2-2.5 liters was present. Left interstitial ectopic pregnancy, which was ruptured posteriorly into the left uterine cornu extending up to the mesosalpinx, was noticed (Figure 3). Left cornual resection with salpingectomy was done, followed by suturing with round body vicryl. Hemostasis was confirmed. The patient underwent multiple blood and blood product transfusions. The postoperative period was uneventful.

## Discussion

Interstitial ectopic pregnancy, although rare, can lead to a catastrophic event. The mortality rate is higher than the other ectopic pregnancies. In this case, failure to diagnose interstitial ectopic is a point of concern. The incidence of interstitial ectopic pregnancy is predisposed if there is a history of any tubal surgery, pelvic inflammatory disease, previous history of ectopic or interstitial pregnancy, infertility, or use of any ovulation-inducing drugs [2]. Interstitial ectopic pregnancy can be confirmed by serial beta-human chorionic gonadotropin (hCG) levels combined with ultrasonography. Ultrasonography will show a vacant cavity of the uterus, a gestational sac (G sac), not in connection with the cavity, and a thin (less than 5 mm) rim of myometrium around the G sac. (It is nothing but a line of echogenicity extending from the cavity up to the corner adjacent to the gestational mass, known as the interstitial line sign [3]).

Interstitial ectopic pregnancy generally ruptures late as compared to other ectopic pregnancies because of the stretching myometrium. Interstitial ectopic pregnancy can be missed on ultrasonography as well. There have been reports of mistaking interstitial pregnancy for intrauterine pregnancy and fruitless attempts at terminating them, finally landing in catastrophic rupture ectopic pregnancy [4]. There are medical and surgical (open or laparoscopic) ways of managing such pregnancies. Managing unruptured interstitial ectopic pregnancy by methotrexate is possible if diagnosed at an early stage. Some study has been done where local injection of methotrexate by ultrasonography (USG) guided into the gestational sac have yielded positive outcome, but the risk of rupture and future recurrence of interstitial or cornual ectopic pregnancy cannot be ruled out [5].

Less invasive procedures such as laparoscopic-assisted transcervical vacuum aspiration and diagnostic hysteroscopy followed by vacuum aspiration can be performed in selected cases [6]. For ruptured cases, earlier hysterectomy or cornual resection was the common mode of management by laparotomy. Laparoscopy has opened options for cornuostomy, cornual resection, or salpingectomy. However, salpingectomy cannot rule out the possibility of recurrent cornual ectopic pregnancy [7]. Some studies have recommended ipsilateral uterine artery ligation before cornual repair as this will help in attaining good hemostasis and achieve better results in a subsequent pregnancy [8]. There have been reports of cornual interstitial ectopic pregnancy after salpingo-oophorectomy of the same side [9,10]. Thus, the previous history of salpingectomy will not prevent the occurrence of ipsilateral cornual interstitial ectopic pregnancy.

## Conclusions

Interstitial ectopic pregnancy has always been a diagnostic and therapeutic challenge. Diagnosing interstitial and cornual pregnancy at the earliest is the key to preventing the morbidity and mortality associated with it. If timely treatment is not received by the patient, morbidity and mortality can be high. In underdeveloped or developing countries with low resource settings, especially in peripheral areas, due to a lack of sonography facilities or lack of availability of investigations, diagnosis can be delayed. So, the clinical abilities of medical professionals should be strong so as not to miss such cases. A high index of suspicion helps in early diagnosis. Patients should be counseled properly regarding follow-up, planning for future pregnancy, and the consequences in a future pregnancy.
